# Building a reliable 16S mini-barcode library of wild bees from Occitania, south-west of France

**DOI:** 10.3897/BDJ.12.e137540

**Published:** 2025-01-07

**Authors:** Anaïs Marquisseau, Kamila Canale-Tabet, Emmanuelle Labarthe, Géraldine Pascal, Christophe Klopp, André Pornon, Nathalie Escaravage, Rémi Rudelle, Alain Vignal, Annie Ouin, Mélodie Ollivier, Magalie Pichon

**Affiliations:** 1 Dynafor, INRAE, INP, ENSAT, 31326, Castanet Tolosan, France Dynafor, INRAE, INP, ENSAT, 31326 Castanet Tolosan France; 2 GenPhySE, Université de Toulouse, INRAE, ENVT, 31326, Castanet Tolosan, France GenPhySE, Université de Toulouse, INRAE, ENVT, 31326 Castanet Tolosan France; 3 MIAT, INRAE, 31326, Castanet Tolosan, France MIAT, INRAE, 31326 Castanet Tolosan France; 4 CRBE, CNRS, UPS, IRD, INP, 31077, Toulouse, France CRBE, CNRS, UPS, IRD, INP, 31077 Toulouse France; 5 Rudelide Expertise muséologie, Rieupeyroux, France Rudelide Expertise muséologie Rieupeyroux France

**Keywords:** wild bees, Apoidea, Anthophila, 16S rRNA, reference database, DNA barcoding

## Abstract

**Background:**

DNA barcoding and metabarcoding are now powerful tools for studying biodiversity and especially the accurate identification of large sample collections belonging to diverse taxonomic groups. Their success depends largely on the taxonomic resolution of the DNA sequences used as barcodes and on the reliability of the reference databases. For wild bees, the barcode sequences coverage is consistently growing in volume, but some incorrect species annotations need to be cared for. The COI (Cytochrome Oxydase subunit 1) gene, the most used in barcoding/metabarcoding of arthropods, suffers from primer bias and difficulties for covering all wild bee species using the classical Folmer primers.

**New information:**

We present here a curated database for a 250 bp mini-barcode region of the 16S rRNA gene, suitable for low-cost metabarcoding wild bees in applications, such as eDNA analysis or for sequencing ancient or degraded DNA. Sequenced specimens were captured in Occitania (south-west of France) and morphologically identified by entomologists, with a total of 530 individuals belonging to 171 species and 19 genera. A customised workflow including distance-tree inferences and a second round of entomologist observations, when necessary, was used for the validation of 348 mini-barcodes covering 148 species. Amongst them, 93 species did not have any 16S reference barcode available before our contribution. This high-quality reference library data are freely available to the scientific community, with the aim of facilitating future large-scale characterisation of wild bee communities in a context of pollinators' decline.

## Introduction

Worldwide, pollinators have become the focus of particular attention as populations decline drastically (Biesmeijer et al. 2006, Rhodes 2018, Powney et al. 2019). Amongst these, wild bees (Hymenoptera, Apoidea, Anthophila) provide the majority of pollination services with more than 20,000 species listed on the Planet (Michener 2007). Many countries have launched important research programmes in order to define actions required for their conservation and restoration, such as ORBIT or RestPoll in Europe. In France, the establishment of the IUCN Red List of wild bees as a part of the current Pollinator Plan (2021-2026) is a major action to counteract the decline of pollinators. In this context, extensive temporal and spatial sampling is crucial for ecological and conservation studies and needs to be associated with rapid and cost-effective identification of large specimens numbers.

Traditionally, arthropod identification, including wild bees, was based on the examination of morphological characters and the time-consuming detection of subtle morphological differences between species requires trained taxonomists. Unfortunately, the lack of policy commitment to training new experts has led to an increasingly intense shortage of specialists, a situation commonly referred to as the taxonomic impediment (de Carvalho et al. 2007, Vinarski 2020). To complement these morphology-based methods, molecular approaches have been developed, rapidly becoming essential tools in modern taxonomy (Chua et al. 2023). In 2007, the Barcode of Life project was initiated in Ontario with the objective of creating a public reference library for all animal species of a standard 640 bp fragment from the mitochondrial COI (Cytochrome Oxydase subunit 1) gene (Ratnasingham and Hebert 2007, Ratnasingham and Hebert 2013). Since then, the COI marker has been widely used for DNA metabarcoding purposes, particularly to describe arthropod biodiversity in various contexts (Piper et al. 2019, Liu et al. 2019, Remmel et al. 2024).

As a consequence, the COI barcode has become the main marker used for cataloguing the genetic diversity of Apoidea Anthophila in many countries worldwide: in Canada (Sheffield et al. 2009, Sheffield et al. 2017), in Chile (Packer and Ruz 2017), in Ireland (Magnacca and Brown 2012), in United Kingdom (Creedy et al. 2020), in Germany (Schmidt et al. 2015), in Luxembourg (Herrera-Mesías et al. 2022), in Slovenia (Janko et al. 2024), in Spain and Portugal (Wood et al. 2024) and in France (Villalta et al. 2021, Ollivier et al. In prep.). However, some of these studies have identified difficulties in efficiently barcoding some wild bee species such as *Andrena* or *Hylaeus* with the classical COI Folmer primers (658 bp) (Folmer et al. 1994, Schmidt et al. 2015, Villalta et al. 2021). An additional difficulty arises from the existence of a few inaccurate wild bees species annotations in the BOLD (Ratnasingham et al. 2024) and GenBank (Clark et al. 2016) databases, such as reported by Herrera-Mesías et al. (2022) and Janko et al. (2024). Thus, for the success of future metabarcoding investigations, there is a need for the evaluation of other barcode sequences and their potential to ensure the largest possible taxonomic coverage of wild bees species and to improve database curation. Amongst the possible candidate mitochondrial genes usable for low-cost wild bee metabarcoding, the 16S rRNA gene could be a good choice because of its short highly variable and conserved regions (Marquina et al. 2018, Elbrecht et al. 2016).

Since over two decades, the 16S locus has already been used to infer the phylogeny of Hymenoptera including bees (Whitfield and Cameron 1998). Molecular phylogenies of Apoidae with 16S rRNA were reported for stingless bees living in Neotropical regions (Costa et al. 2003, Trianto and Purwanto 2020, Marconi et al. 2023) and for honey bees subspecies in Saudi Arabia (Alajmi et al. 2019). With primers derived from the *Apismellifera* 16S sequences, the phylogeny of some Korean bumblebees was clarified (Yoon et al. 2004). Kek et al. (2017) tested two short regions of both the COI and 16S genes to discriminate bee species involved in honey production and demonstrated that a 287 bp region of the 16S rRNA gene was more informative than a 284 bp region of the COI gene. Moreover, a 16S mini-barcode (120 bp long) has been tested with success on Insecta class by Hsieh et al. (2019).

Targeting a short barcode gene region (hereafter referred to as mini-barcode) is particularly interesting for approaches requiring to overcome DNA degradation, while preserving a high level of taxonomic resolution (Hajibabaei et al. 2006). Amongst these approaches, sequencing museum specimens (Levesque-Beaudin et al. 2023, Santos et al. 2023) or eDNA based biomonitoring provide encouraging prospects (Newton et al. 2023, Sickel et al. 2023, Avalos et al. 2024). Currently, there is no mini-barcode library available for wild bee species, unlike the ones already available for plants (Little 2014) and marine macrophytes (Ortega et al. 2020).

In this study, our main objectives were to evaluate the 16S mini-barcode potential (Clarke et al. 2014) to discriminate wild bee species and build a robust database facilitating future DNA metabarcoding investigations on these important pollinators.

## Sampling methods

### Step description

**Collection description**

The 530 specimens used in this study originated from three sources: 1) 412 from the UMR DYNAFOR collection stored at INP-ENSAT (Ollivier et al. 2024); 2) 88 from the private collection of the bee expert, Rémi Rudelle and 3) a set of 30 *Bombus* specimens from the CRBE (Centre de Recherche sur la Biodiversité et l‘Environnement) collection. Metadata with detailed information related to each specimen (geographic location, identifiers, sex etc.) can be found in Suppl. material 1.

**1. Sample collection**

For the UMR DYNAFOR collection, three coloured pan traps (blue, white and yellow) were set in the grassy strip boarding the crop. Each pan trap contained 250 ml of water with Teepol (3 drops/l). After 3 days of exposure, the insects were filtered and placed in ethanol (EtOH) 70% until identification. A panel of 61 specimens was captured with nets and euthanised in ethyl acetate vapour (Suppl. material 1). For the private collection of Rémi Rudelle and the CRBE collection, the specimens were captured with nets.

All of the specimens were morphologically identified using mostly the Insecta Fauna Helvetica reference (Amiet 1996, Amiet 1999, Amiet et al. 2001, Amiet et al. 2004, Amiet et al. 2007, Amiet et al. 2010, Amiet et al. 2017) and others (Schmid-Egger and Scheuchl (1997), Wood (2023) for the Andrenidae family; Ortiz-Sanchez and Jimenez-Rodriguez (1991), Terzo et al. (2007), Rasmont (2014), Smit (2018), Aubert (2020), Le Divelec (2021), Rasmont et al. (2021) for the Apidae family; Ornosa and Ortiz-Sanchez (2004) for the Colletidae family; Pauly (2019) for the Halictidae family and Benoist (1931), Benoist (1941), Pauly (2015) for the Megachilidae family) by one of the following entomologists: Rémi Rudelle, David Genoud, Romain Carrié, Léa Frontero and Dominique Pelletier. They are conserved, pinned in insect boxes and stored at room temperature. To prevent parasite infestation, specimens are frozen twice a year at -20°C for at least 48 hours for the UMR DYNAFOR collection.

**2. Sequencing and processing**

DNA was extracted from a portion or an entire leg of dried specimens using the Chelex method (see Casquet et al. (2012) for a detailed protocol). Two sequencing technologies were used: 275 specimens were sequenced using high-throughput Illumina technology (MiSeq Sequencing System) and 255 were sequenced with Sanger technology. The list of all species with the corresponding sequencing method is included in Suppl. material 1.

MiSeq sequencing and processing

For the set of 275 specimens processed with MiSeq sequencing, two microlitres of DNA were used as template for PCR. 16S primers ins16S_1R/ins16S_1F (R: TRRGACGAGAAGACCCTATA; F: TCTTAATCCAACATCGAGGTC, Clarke et al. (2014)) were chosen to amplify a 250 bp region of the mitochondrial 16S gene. PCR was performed in a 20 µl total volume containing 5.84 µl of purified water, 10 µl of 2x ampliTaq Gold 360 master mix (Thermo Scientific LSG Life Technologies) including dNTP and ampliTaq Gold, 0.16 µl BSA, 1 µl of each primer (5 µM) and 2 µl of DNA. PCR was carried out under the following conditions: hot-start at 95°C for 10 min followed by 40 cycles (denaturation at 95°C for 30 s, primer annealing at 49°C for 30 s and primer extension at 72°C for 30 s); and final extension at 72°C for 7 min. Primers were 5′ labelled with a set of 8 bp tags to identify samples in bioinformatics analysis. 16S PCR products were visualised on 1% TAE agarose gels quantified using PicoGreen dsDNA Quantitation Reagent and mixed aiming at equimolar pools. The pool was then purified using beads contained in the Illumina TruSeq Nano kit (part #15041758) and libraries were generated following the manufacturer’s guide for the Illumina TruSeq Nano kit, except that no sonication was performed. Libraries were sequenced on a single run of an Illumina MiSeq (2 × 250 paired-end reads), using the NGS core facility at the Génopole Toulouse Midi-Pyrénées. We obtained 22,540,200 demultiplexed reads (R1 and R2 reads). 16S rDNA amplicon sequences were analysed using the FROGS pipeline (version 3.1, Escudié et al. (2017)). Amplicons were processed according to their size (150 - 490 nucleotides) and clustered into ASVs (Amplicon Sequence Variant) using Swarm (aggregation distance: d = 1) (Mahé et al. 2014). For each sample, the most abundant ASV was kept for the procedure of barcode validation.

Sanger sequencing and processing

**Figure 1.**
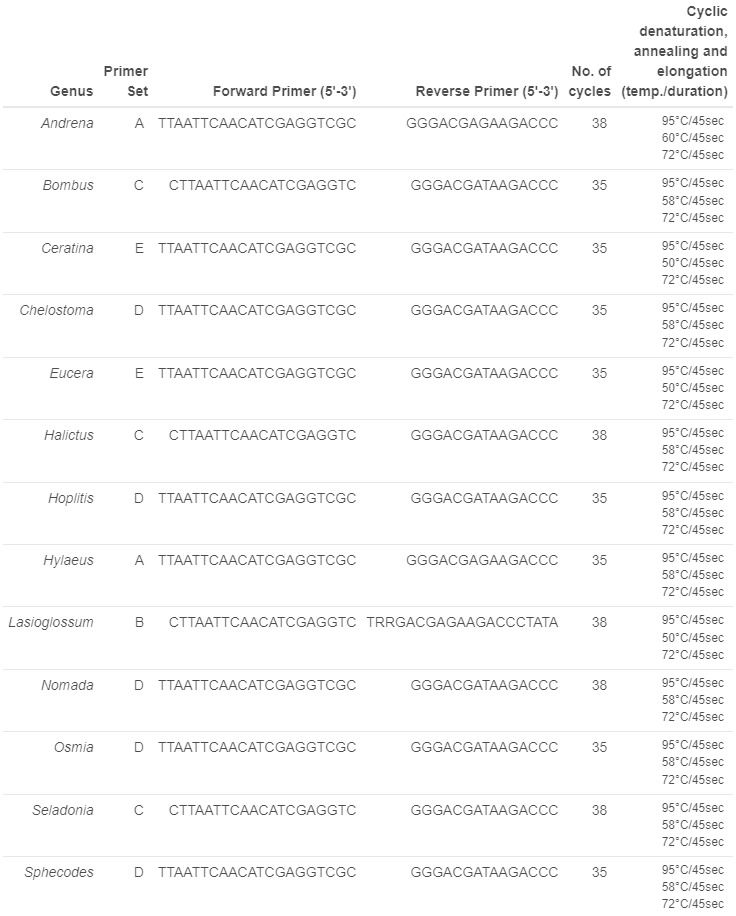
**Primer sequences and PCR conditions used for Sanger sequencing per Genus.** For all Sanger samples, primary denaturation was performed at 95°C for 5 minutes and final elongation was performed at 72°C for 20 minutes.

DNA barcoding using Sanger sequencing technology was performed on 255 specimens. Specific primers were used for each genus. All primer sequences and PCR conditions are given in Fig. 1. For each PCR reaction, 3 µl of extracted DNA was amplified in 25 µl final volume, 1 µM for each primer, 1 x PCRBIO Reaction Buffer (including Mg and dNTPs) and 0.25 µl of PCRBIO Taq DNA Polymerase (5 u/μl) (PB10.11-20; Eurobio). Prior to sequencing, a volume of 2.5 µl from each PCR product was examined on a 2% agarose gel electrophoresis to check the success and specificity of the PCR amplification. The sequencing reaction was subsequently prepared as follows: 2.5 µl of each PCR product was purified by adding 1 µl of each Exonuclease (M0293L; Ozyme) and TSAP (Thermo Sensitive Alcaline Phosphatase) (M9910; Promega) in a final volume of 18 µl. The sequencing reaction mixture was split in two volumes and 1 µl of 10 mM of each primer (forward and reverse) was added. PCR products were sent to a private company for Sanger sequencing in both directions. The sequences produced were manually checked for base calling using ChromasPro 2.1.10.1. (Technelysium Pty Ltd, Tewantin, Australia) and unreadable sequences were removed. For the 30 *Bombus* sequences from CRBE laboratory, the PCR was performed with the forward primer: CGCTGTTATCCCTAAGG and the reverse primer CTGTACAAAGGTAGCATAATC.

**Figure 2.**
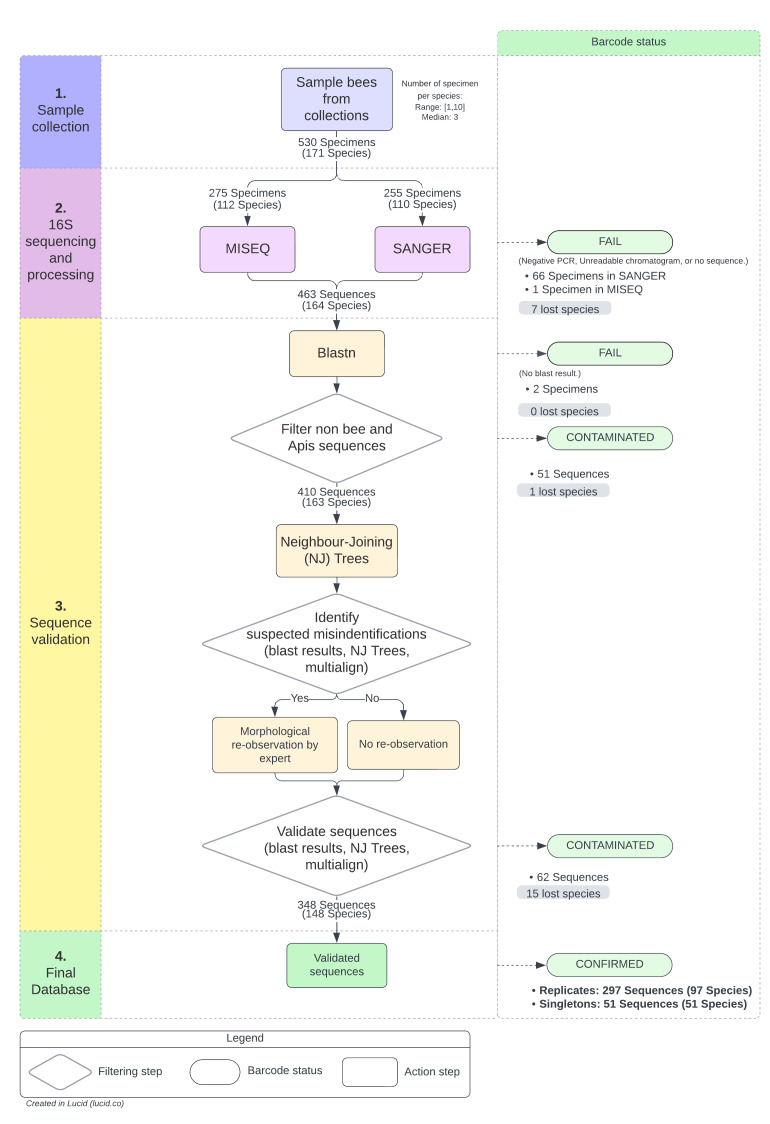
16S mini-barcode library workflow, from sampling to validation. There are four main steps represented by different colours in the left panel, from top to bottom. The middle part of the figure represent the workflow. The right panel indicates the final barcode status. For sequence validation, we used : sequence assignation by BLASTn (Altschul et al. 1990) on GenBank nt database (Sayers et al. 2021); Neighbour-joining (NJ) distance-tree inferences using the K2P model and the Muscle algorithm (Edgar 2004) for alignment implemented in the BOLD toolkit; Sequence alignment using multalin software to visualise allelic variations (Corpet 1988). For some species, the default BLAST parameters were adjusted to take into account for the high AT content of the region in this genus.

Amongst the 171 species included in our study, 43 were represented by a single specimen, 25 by two specimens, 51 by three specimens and 52 by four to ten specimens, corresponding to a total of 530 specimens (Fig. 2, step 1).

The global success rate after MiSeq sequencing was very high, reaching 99%, with only a single specimen failing. The sequencing success with the Sanger technology was lower due to negative PCR or unreadable chromatograms. Indeed, no sequence could be obtained for 66 specimens. However, as replicate samples were included for most species, only seven species were excluded (Suppl. material 3) at this step (Fig. 2, step 2).

**3. Sequence validation**

The 463 sequences corresponding to the remaining 164 species were searched in GenBank (nt database) using BLASTn (Altschul et al. 1990) and contaminants (sequences which do not match with a wild bee reference) were removed from data. Fifty-three non-bee and *Apis* sequences were eliminated and the 410 remaining sequences (163 species) were analysed by cross validation with two filtering rounds (Fig. 2, step 3). The first round allows the detection of potential misidentifications as incongruence between the morphologically and the genetically-based species identification through Neighbour-Joining Trees. For species with only one specimen (singleton), the barcode was validated if the sequence belonged to the corresponding genus. Potentially misidentified species, as well as species that are known to be part of a species complex, were submitted to an entomologist for a second observation taking into account possible identification key updates. The second filtering round allowed sequences validation and confirmation as accurate barcodes. As a result, 16 additional species were excluded (Suppl. material 3).

In total 348 sequenced samples corresponding to 148 unique species were successfully analysed and validated and 97 species were represented by at least two specimens (Fig. 2, step 4).

## Geographic coverage

### Description

**Figure 3.**
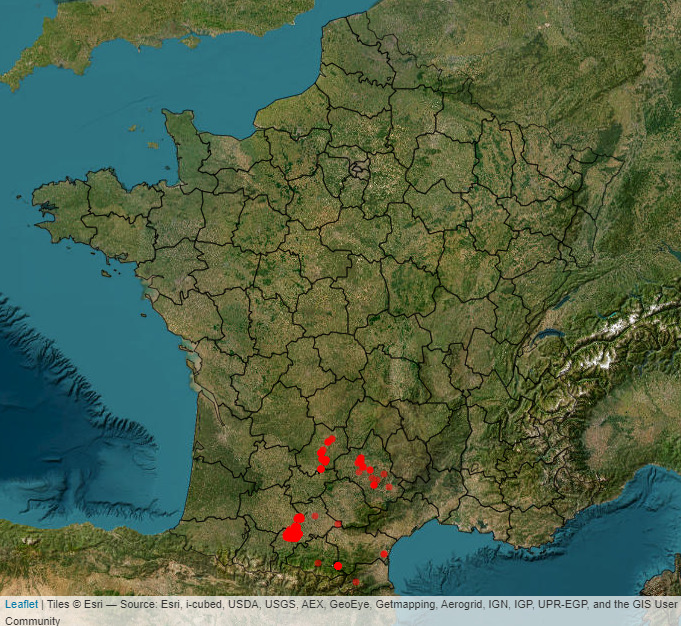
Geographic distribution of the 16S database specimens collected in Occitanie (red points). Nine specimens are not mapped as they were collected outside of Occitanie, although they represent species that can be found within the region.

The wild bees presented in this study were collected from the French region of Occitanie (Fig. 3). The 412 specimens coming from the UMR DYNAFOR collection were collected in 17 sites located in south-west of France, in the long term socio-ecological research site Zone Atelier Pyrénées-Garonne (ZA PYGAR, Ouin et al. (2021)) over a period of 7 years (2013-2019). The ZA PYGAR takes place in the Pyrénées foothills and is characterised by a mosaic of landscapes with crops and small forests (Carrié et al. 2018, Rivers-Moore et al. 2020). Eighty-eight specimens were captured by Rémi Rudelle in different sites of the Aveyron, French Department and 30 *Bombus* specimens from the CRBE collection were sampled in the Pyrénées Orientales, French Department (personal communication Nathalie Escaravage).

## Taxonomic coverage

### Description

**Figure 4.**
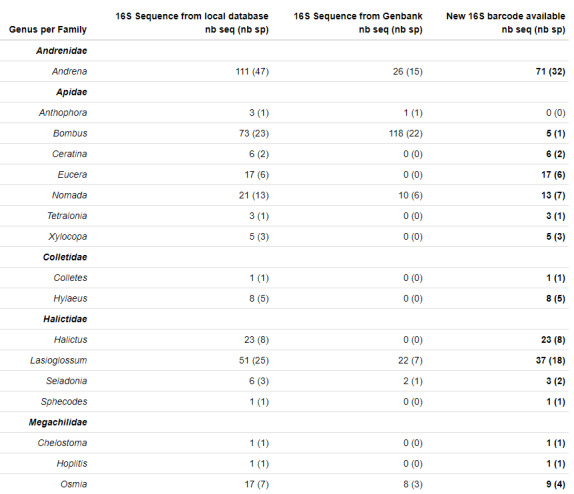
Taxonomic coverage of the 16S mini-barcode library compared to existing sequences in GenBank. nb seq: Number of sequences ; nb spe: Number of species. As a point of reference, the sequences available on the public database GenBank before the project start are given in the second column (only for species recorded in the area of collection ZA PYGAR). The last column indicates the number of new sequences added in GenBank and BOLD. Number of species corresponding to specimen records are indicated in brackets.

Specimen records are reported for the 348 sequences (148 species) confirmed with the above workflow. Fig. 4 displays the sequences and species covered by the herein presented 16S library for each genus.

For 55 of the species included in our dataset, 16S partial or full-length sequences were already available in GenBank (Fig. 4). The list and the accession number of these is reported in Suppl. material 4. The *Bombus* genus is the most represented with 22 species and 118 sequences. For *Andrena*, 26 16S sequences corresponding to 15 species originated from the mitochondrion sequencing project were available. Seven species (22 sequences) of *Lasioglossum* were available at the time of writing the manuscript. At the end, there were no 16S data for 10 genera of wild bees. Thus, we provide 204 new 16S mini-barcodes for wild bees belonging to 93 species. For the most abundant species of France belonging to the *Andrena* and *Lasioglossum* genera sets, 71 new sequences (32 species) and 37 new sequences (18 species) were respectively added in the public databases.

## Temporal coverage

**Data range:** 2010-5-18 – 2020-7-22.

## Usage licence

### Usage licence

Other

### IP rights notes

CC BY 4.0

## Data resources

### Data package title

16S mini-barcode library of wild bees from Occitania

### Resource link

dx.doi.org/10.5883/DS-BCWBS16S

### Number of data sets

1

### Data set 1.

#### Data set name

DS-BCWBS16S

#### Data format

tsv, fasta

#### Download URL

https://v4.boldsystems.org/index.php/Public_BINSearch?searchtype=records

#### Description

The list of the 530 specimens (171 species) with complementary information such as their BOLD IDs, process IDs, GenBank IDs (only for sequences > 200 bp), taxonomy, identifiers, gps location for UMR DYNAFOR collection, sequencing method, barcode status (failed, contaminated or confirmed replicate/single) is contained in the dataset. It covers five families, 19 genera and 171 species. After sequencing and validation barcode steps, 348 sequences corresponding to 148 species and 17 genera were selected. Suppl. materials 1, 2 can be downloaded as the version of the dataset (metadata and fasta sequences) at the time of writing the manuscript.

**Data set 1.** 

Column label	Column description
Sample_ID	Unique BOLD identifier for the specimen.
Process_ID	Unique BOLD identifier for the barcode.
Accession_NCBI	Unique GenBank identifier for the barcode (Accession number).
Museum_ID	Unique collection identifier for the specimen.
Collection_code	Identifier for the collection: Dynafor, RIEUPEYROUX or CRBE.
Institution_storing	Institution where specimens are physically stored: ENSAT, Rudélide Expertise Muséologie REM or CNRS.
Phylum	Phylum name
Class	Class name
Order	Order name
Family	Family name
Subfamily	Subfamily name
Genus	Genus name
Species	Species name
Subspecies	Subspecies name
Identifier	Name of the individual who identified the specimen morphologically.
Identifier_Email	Email of the identifier.
Identification_Method	All specimens were morphologically identified.
Sex	The sex of the specimen: F for female, M for male.
Specimen's caste	Extra information about the specimen's caste: W if the specimen is a worker (empty otherwise).
Life_stage	Life stage of the sampled specimen. All specimens were adults.
Tissue_descriptor	Type of tissue analysed: LEG.
Collectors	Names of the individuals who collected the specimen in the field.
Collection_Date	Exact date during which the specimen was collected. For CRBE specimens, only the collection year is available.
Country	Name of the country in which the specimen was collected. All specimens were collected in France.
State	Name of the state (French: Région) in which the specimen was collected. All the specimens, except nine, were collected in Occitanie.
Region	Name of the region (French: Département) in which the specimen was collected.
Sector	Name of the sector or city, in which the specimen was collected.
Exact_Site	A brief description of the site in which the specimen was collected.
Latitude	The geographic latitude (in decimal degrees) of the site in which the specimen was collected. For CRBE or Rudélide specimens, only an approximate latitude is available, corresponding to the latitude of the municipality rather than the exact collection point.
Longitude	The geographic longitude (in decimal degrees) of the site in which the specimen was collected. For CRBE or Rudélide specimens, only an approximate longitude is available, corresponding to the longitude of the municipality rather than the exact collection point.
Sampling_protocol	The sampling method used to capture the specimen: NET or PAN TRAP.
Sequencing_method	The method used to sequence the specimen: SANGER or MISEQ.
Barcode_status	The status of the 16S barcode for the specimen: Confirmed_single, Confirmed_replicate, Contaminated or Fail.

## Additional information

### Sequencing and barcoding results

**Figure 5.**
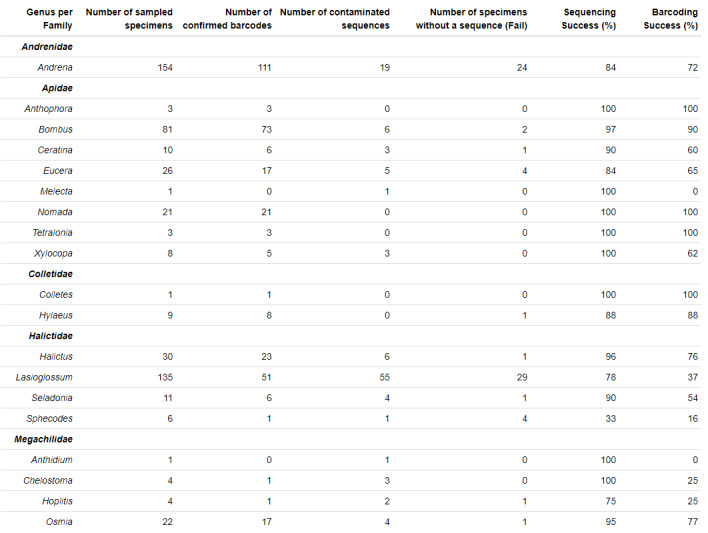
Sequencing and barcoding success per family and genus.

The within-genus global sequencing success including MiSeq and Sanger technologies varies from 75% to 100%, except for *Sphecodes* (33%) (Fig. 5). For the most represented genus of our dataset, namely *Andrena* and *Lasioglossum*, the sequencing success rates were 84% and 78%, respectively. The barcoding success rate after all successive filtering steps was different according to genera. It was 100% for *Nomada*, *Tetralonia*, *Antophora* and *Colletes*, 90% for *Bombus*, 88% for *Hylaeus*, 77% for *Osmia*, 76% for *Halictus*, 72% for *Andrena* and it was under 65% for the other genera. For *Lasioglossum*, the low barcoding success rate (38%) is related to low sequencing success, being as low as 42.7% with MiSeq and 28% with Sanger (Suppl. material 1) and to high level of contaminated sequences (54). An in-depth analysis of *Lasioglossum* sequences showed that the end of the amplified fragment contains many stretches of AT nucleotide repeats (Suppl. material 2) which are known to disrupt the polymerase activity during the sequencing process. Amongst the 171 species of our dataset, no barcode could be obtained for 23 species including nine species of *Lasioglossum* (Suppl. material 3). Suppl. material 5 provides detailed sequencing and barcoding success per species.

### Analysis of genetic distances

**Figure 6.**
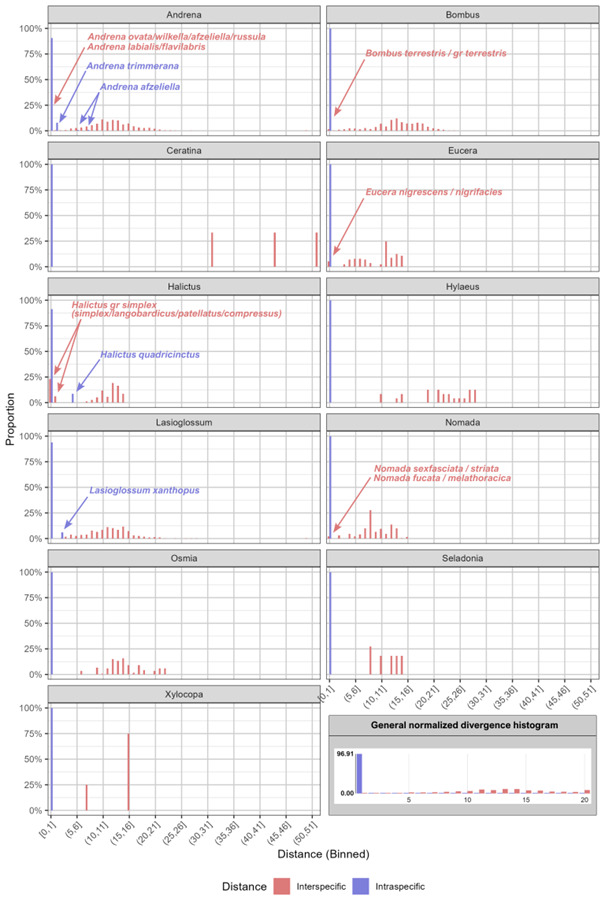
Distribution of intraspecific and interspecific genetic distances per genus. The global normalised distance distribution for all specimens is shown at bottom right corner. Blue arrows indicate species that show an intraspecific distance > 1%. Red arrows indicate group of species that show an interspecific distance < 1%.

Examination of the general normalised divergence histogram performed with BOLD analyses toolkit on all species (replicates) indicates the existence of a DNA barcoding gap (maximal intraspecific distance > minimal interspecific distance), allowing reliable molecular identification of specimens (Fig. 6). However, a more in-depth analysis of each genus reveals two scenarios: There was an overlap between intraspecific and interspecific genetic distances in six genera: *Andrena*, *Bombus*, *Eucera*, *Halictus*, *Lasioglossum* and *Nomada*, whereas the barcoding gap was clearly existing for the five others genera: *Xylocopa*, *Seladonia*, *Hylaeus*, *Osmia* and *Ceratina* (Fig. 6). The tables with detailed intraspecific and interspecific genetic distances are given in Suppl. materials 6, 7.

**Genetic distance analyses per family and genus**

- **Andrenidae**: (Taxon ID tree is given in Suppl. material 8).

For the *Andrena* genus, which has been reported difficult to barcode with the COI Folmer primers (Schmidt et al. 2015, Villalta et al. 2021), the 16S mini-barcode offers a good alternative. Amongst the 111 validated barcodes (47 species), the 16S mini-barcode allows us to discriminate all the species in accordance with the morphological subgenera classification (Suppl. material 8). Interestingly, the 250 bp of the 16S gene used in this study is sufficiently divergent to separate complex groups previously described in literature. For example, the barcoding of *Andrenadistinguenda* species group (*A.nitidula* and *A.distinguenda*) with COI showed the existence of two bins (Schmidt et al. 2015). With the 16S mini-barcode, the minimum divergence between these two species was 1.13% supporting the existence of two species (Suppl. material 7). Similar to that described by Wood et al. (2021), we found a clear separation (3.11% minimum divergence) in the *Andrenaangustior* group between *Andrenaimpressa* and *Andrenafulvata* (Suppl. material 7). A complex situation remains with *Andrenatrimmerana*; our molecular data on eight specimens including two males and six females clearly show two groups (0% divergence within groups) with an intergroup divergence of 1.29% (Suppl. material 7). Interestingly, we observed allelic variation (1 SNP) between *Andrenadorsata* originating from our data and the two *Andrenadorsata* sequences provided in GenBank originated from the UK (KT16433.1 and OV815490.1). Elsewhere, two sequences of *Andrenafulva* were available in GenBank (KT164623.1 and OX276334.1). Alignment of these two *A.fulva* with our specimens reviewed by entomologists show that KT16423.1 is 100% homologous with our *Andrenafulvago*, whereas OX276334.1 aligns with *Andrenafulva*. The sequences of these two species diverge 5.9% with the 16S mini-barcode.

- **Apidae**: (Taxon ID tree is given in Suppl. material 9).

***Bombus***: The distance tree inferred from the 16S mini-barcodes of the 23 *Bombus* species (73 specimens) reveals genetic divergence that is consistent with the known subgenus classification (Cameron et al. 2007, Cejas et al. 2019, Sun et al. 2021). Interestingly, the 16S mini-barcode classifies without ambiguity each specimen of the following species complex: *Bombuspascuorum*/*muscorum*; *Bombussylvarum*/*ruderarius*; *Bombusruderatus*/*hortorum*; *Bombuspyreaneus*/*pratorum* and *Bombusterrestris*/*lucorum*. As some of the specimens in the *Bombusterrestris* group in our dataset were not morphologically identified at the species level, the 0% divergence between specimens named Bombusgr.terrestris and *Bombusterrestris* suggests that all of them are *Bombusterrestris*. Elsewhere, amongst the 10 *Bombuspascuorum* specimens barcoded with the 16S mini-barcode, some allelic variation was observed, with a minimum intraspecific divergence of 0% and a maximum intraspecific divergence of 0.98% (Suppl. material 6). In Switzerland, Amiet et al. (2017) reported the presence of two subspecies *of Bombuspascuorum*.

***Eucera***: In the Apidae family, the 16S mini-barcodes are not discriminant for two species belonging to the *Eucera* genus: *Euceranigrifacies* and *Euceranigrescens*, whereas it efficiently delineates the six others species, especially *Euceralongicornis* which was confused in the past with *Euceranigrescens* (Dorchin et al. 2018). Allelic variations are observed for *Euceralongicornis* (0.26% maximum intraspecific divergence) and *Euceranumida* (0.26% maximum intraspecific divergence) (Suppl. material 6).

***Nomada***: Regarding the *Nomada* genus, recently reexamined by Odanaka et al. (2022) and Straka et al. (2024), our data shows that the 16S mini-barcode distinguishes the 21 specimens (13 species), except *Nomadastriata* versus *Nomadasexfasciata* and *Nomadafucata* versus *Nomadamelathoracica*. However, more specimens need to be analysed to conclude definitively that three of these species are singletons.

***Ceratina***: The molecular phylogeny of these small carpenter bees has been recently achieved by Sless et al. (2024). The two species of our dataset are extremely divergent (31.12% min interspecific divergence and 51.49% max interspecific divergence, Suppl. material 7). One belongs to the subgenus Euceratina (*Euceracyanea*) and the other to the subgenus Ceratinasensustricto (*Ceratinacucurbitinia*).

***Xylocopa***: The five specimens of *Xylocopa* from our dataset correspond to the three species: *Xylocopavalga*, *Xylocopairis* and *Xylocopaviolacea* exhibit 0.46% maximum intraspecific divergence and 7.19% minimum interspecific divergence (Suppl. materials 6, 7).

- **Colletidae** (Taxon ID tree is given in Suppl. material 10).

In the present study, five species belonging to *Hylaeus* (Almeida and Danforth 2009) genus were successfully barcoded with the 16S mini-barcode. Amongst them, four were singletons and *Hylaeusbrevicornis* had three replicates with intraspecific divergence of 0% (Suppl. material 6). The divergence between species ranges from 10.47% to 28.66% (Suppl. material 7).

- **Megachilidae** (Taxon ID tree is given in Suppl. material 11).

***Osmia***: As *Apismellifera* or *Bombus*, *Osmia* are commercially reared for pollination services. A complete phylogeny of the Palearctic Osmiine bee is available on the website of Müller (2024). Molecular data using UCEs or Elongation factor 1-α or LW-rhodopsin and Conserved ATPase domain have been reported by Praz et al. (2008) and Branstetter et al. (2021). In this work, the 16S mini-barcode is clearly efficient to separate the seven species of our dataset: 0.51% maximum intraspecific divergence and 6.22% minimum interspecific divergence were observed (Suppl. materials 6, 7).

- **Halictidae** (Taxon ID tree is given in Suppl. material 12).

***Lasioglossum***: Molecular phylogeny of *Lasioglossum* is poorly documented (Danforth 1999, Gibbs et al. 2012, Gibbs 2018, Pauly et al. 2019). For the 25 species belonging to genus *Lasioglossum* successfully barcoded in this study, the intraspecific divergence is < 1% for all species, except for *Lasioglossumxanthopus* which is > 2%. Interestingly, the 16S mini-barcode allows a clear separation (min interspecific divergence > 2%) for complex groups. Thus, for the specimens of *Lasioglossummedinai*/*Lasioglossumvillosulum* species, the min interspecific divergence is 4.38%. It is 6.84% for *Lasioglossummalachurum*/*subhirtum*/*calceatum*/*pauxillum*/*laticeps* species; 5.70% for *Lasioglossummorio*/*nitidulum* species and 5.23% for *Lasioglossumpauperatum*/*pygmaeum*/*truncaticolle*/*crassepunctatum* species (Suppl. materials 6, 7).

***Halictus***: In the *Halictussimplex* group, *Halictussimplex* and *Halictuslangobardicus* females are extremely difficult to distinguish morphologically. Unfortunately, the 16S mini-barcode did not allow for the discrimination of the two species, whereas *Halictuscompressus* exhibited a 0.78% minimum interspecific divergence with the rest of the group (Suppl. material 7). Interestingly, two specimens from the *Halictussimplex* group diverged slightly from the others. It would be interesting to barcode more specimens including species belonging to the simplex group which were not included in our dataset. Sequencing complete mitochondrion in the future would help to clarify the status of this group. Elsewhere, we observed allelic variation amongst specimens of *Halictusquadricinctus* and we suspected that one of them could be *Halictusbrunnescens*.

### Concluding remarks

The 250 bp 16S mini-barcode used in this study allows wild bee identification of all species, except two specimens of the *Melecta* and *Anthidium* genus. Integrative approaches coupling examination of distance trees, multiple alignment and comparison with morphological data allowed us: 1) to provide 204 new 16S mini-barcodes for wild bees belonging to 93 species verified by taxonomists; 2) to identify species complexes and 3) to delineate efficiently species when females were difficult to separate. This opens avenues for the 16S mini-barcode to be used as an efficient and reliable additional marker in the toolkit for anyone relying on molecular technologies for wild bees ecological studies.

## Supplementary Material

D59CE6D3-110E-5940-B7F4-4459AAA0F5C0 10.3897/BDJ.12.e137540.suppl1 Supplementary material 1 16S Wild Bees mini-barcodes from Occitania: metadata Data type Metadata File: oo_1182964.tsv https://binary.pensoft.net/file/1182964 Anaïs Marquisseau, Kamila Canale-Tabet, Emmanuelle Labarthe, Géraldine Pascal, Christophe Klopp, André Pornon, Nathalie Escaravage, Rémi Rudelle, Alain Vignal, Annie Ouin, Mélodie Ollivier, Magalie Pichon

7956E49A-155D-5277-BE33-29F3421AA288 10.3897/BDJ.12.e137540.suppl2 Supplementary material 2 16S Wild Bees mini-barcodes from Occitania: DNA sequences Data type DNA Sequences, Fasta File: oo_1107016.fasta https://binary.pensoft.net/file/1107016 Anaïs Marquisseau, Kamila Canale-Tabet, Emmanuelle Labarthe, Géraldine Pascal, Christophe Klopp, André Pornon, Nathalie Escaravage, Rémi Rudelle, Alain Vignal, Annie Ouin, Mélodie Ollivier, Magalie Pichon

7378EF15-C888-5FF2-B0BB-C26D9ED36FFD 10.3897/BDJ.12.e137540.suppl3 Supplementary material 3 Lost species Data type List Brief description List of the 23 species eliminated during the barcode acquisition process. File: oo_1107025.tsv https://binary.pensoft.net/file/1107025 Anaïs Marquisseau, Kamila Canale-Tabet, Emmanuelle Labarthe, Géraldine Pascal, Christophe Klopp, André Pornon, Nathalie Escaravage, Rémi Rudelle, Alain Vignal, Annie Ouin, Mélodie Ollivier, Magalie Pichon

FB066A98-AF0D-5B7B-8A86-93D4227A275F 10.3897/BDJ.12.e137540.suppl4 Supplementary material 4 Available 16S in GenBank Data type List, Accession numbers File: oo_1107028.tsv https://binary.pensoft.net/file/1107028 Anaïs Marquisseau, Kamila Canale-Tabet, Emmanuelle Labarthe, Géraldine Pascal, Christophe Klopp, André Pornon, Nathalie Escaravage, Rémi Rudelle, Alain Vignal, Annie Ouin, Mélodie Ollivier, Magalie Pichon

794B51C4-1060-5AB7-83A4-90088CF1CDFC 10.3897/BDJ.12.e137540.suppl5 Supplementary material 5 Sequencing and barcoding success per species Data type Occurrences File: oo_1107023.tsv https://binary.pensoft.net/file/1107023 Anaïs Marquisseau, Kamila Canale-Tabet, Emmanuelle Labarthe, Géraldine Pascal, Christophe Klopp, André Pornon, Nathalie Escaravage, Rémi Rudelle, Alain Vignal, Annie Ouin, Mélodie Ollivier, Magalie Pichon

78B68B87-96DD-550A-8881-023D627C5048 10.3897/BDJ.12.e137540.suppl6 Supplementary material 6 Table of intraspecific distances Data type Genetic distances Brief description Distances between specimens within species. To find minimum and maximum intraspecific distance for a specific species, filter the column "Species". File: oo_1182965.txt https://binary.pensoft.net/file/1182965 Anaïs Marquisseau, Kamila Canale-Tabet, Emmanuelle Labarthe, Géraldine Pascal, Christophe Klopp, André Pornon, Nathalie Escaravage, Rémi Rudelle, Alain Vignal, Annie Ouin, Mélodie Ollivier, Magalie Pichon

1F1E8687-64EB-56C7-A574-D28F42C40C46 10.3897/BDJ.12.e137540.suppl7 Supplementary material 7 Table of interspecific distances Data type Genetic distances Brief description Distances between specimens belonging to different species within their genus. To find minimum and maximum interspecific distance between two species, filter the columns "species_1" and "species_2". To find minimum and maximum intragenus distance, filter the column "Genus". File: oo_1182946.txt https://binary.pensoft.net/file/1182946 Anaïs Marquisseau, Kamila Canale-Tabet, Emmanuelle Labarthe, Géraldine Pascal, Christophe Klopp, André Pornon, Nathalie Escaravage, Rémi Rudelle, Alain Vignal, Annie Ouin, Mélodie Ollivier, Magalie Pichon

1754F3D5-2AA6-5F03-AC3A-F55FBC29A5E7 10.3897/BDJ.12.e137540.suppl8 Supplementary material 8 Andrenidae Tree Data type NJ Tree File: oo_1135512.pdf https://binary.pensoft.net/file/1135512 Anaïs Marquisseau, Kamila Canale-Tabet, Emmanuelle Labarthe, Géraldine Pascal, Christophe Klopp, André Pornon, Nathalie Escaravage, Rémi Rudelle, Alain Vignal, Annie Ouin, Mélodie Ollivier, Magalie Pichon

397E3B69-14B1-56D3-BB7D-05D224625834 10.3897/BDJ.12.e137540.suppl9 Supplementary material 9 Apidae Tree Data type NJ Tree File: oo_1135511.pdf https://binary.pensoft.net/file/1135511 Anaïs Marquisseau, Kamila Canale-Tabet, Emmanuelle Labarthe, Géraldine Pascal, Christophe Klopp, André Pornon, Nathalie Escaravage, Rémi Rudelle, Alain Vignal, Annie Ouin, Mélodie Ollivier, Magalie Pichon

B087F177-D2CF-5EDD-A691-AF8594015A67 10.3897/BDJ.12.e137540.suppl10 Supplementary material 10 Colletidae Tree Data type NJ Tree File: oo_1128832.pdf https://binary.pensoft.net/file/1128832 Anaïs Marquisseau, Kamila Canale-Tabet, Emmanuelle Labarthe, Géraldine Pascal, Christophe Klopp, André Pornon, Nathalie Escaravage, Rémi Rudelle, Alain Vignal, Annie Ouin, Mélodie Ollivier, Magalie Pichon

AFAE499D-E826-5363-B6F5-D10D5CB32A0D 10.3897/BDJ.12.e137540.suppl11 Supplementary material 11 Megachilidae Tree Data type NJ Tree File: oo_1128834.pdf https://binary.pensoft.net/file/1128834 Anaïs Marquisseau, Kamila Canale-Tabet, Emmanuelle Labarthe, Géraldine Pascal, Christophe Klopp, André Pornon, Nathalie Escaravage, Rémi Rudelle, Alain Vignal, Annie Ouin, Mélodie Ollivier, Magalie Pichon

1B945ADB-43D7-549E-A241-856348E6773B 10.3897/BDJ.12.e137540.suppl12 Supplementary material 12 Halictidae Tree Data type NJ Tree File: oo_1128833.pdf https://binary.pensoft.net/file/1128833 Anaïs Marquisseau, Kamila Canale-Tabet, Emmanuelle Labarthe, Géraldine Pascal, Christophe Klopp, André Pornon, Nathalie Escaravage, Rémi Rudelle, Alain Vignal, Annie Ouin, Mélodie Ollivier, Magalie Pichon
