# Supplementary material for: Building a reliable 16S mini-barcode library of wild bees from Occitania, south-west of France
Source: Biodivers Data J. 2025 Jan 7;13:e137540. doi: 10.3897/BDJ.12.e137540 (PMC11733625; doi:10.3897/BDJ.12.e137540)
Supplement: Supplementary material 8 — Andrenidae Tree [file bdj-13-e137540-s008.pdf]

BOLD TaxonID Tree

Title	:	Tree Result - DS-ANDR16
Date	:	03-Sep-2024
Data Type	:	Nucleotide
Distance Model	:	Kimura 2 Parameter
Marker	:	16S
Colourization	:	[blue]=Stop Codons [red]=Contamination or misidentification
Label	:	Sample ID
Label	:	Taxon
Label	:	Sex/Gender
Label	:	Sequence Length
Sequence Count	:	110
Species count	:	46
Genus count	:	1
Family count	:	1
Unidentified	:	0
BIN Count	:	0

Legend :

- Allelic variant
- Non discriminant 16S
- Discriminant 16S for group species

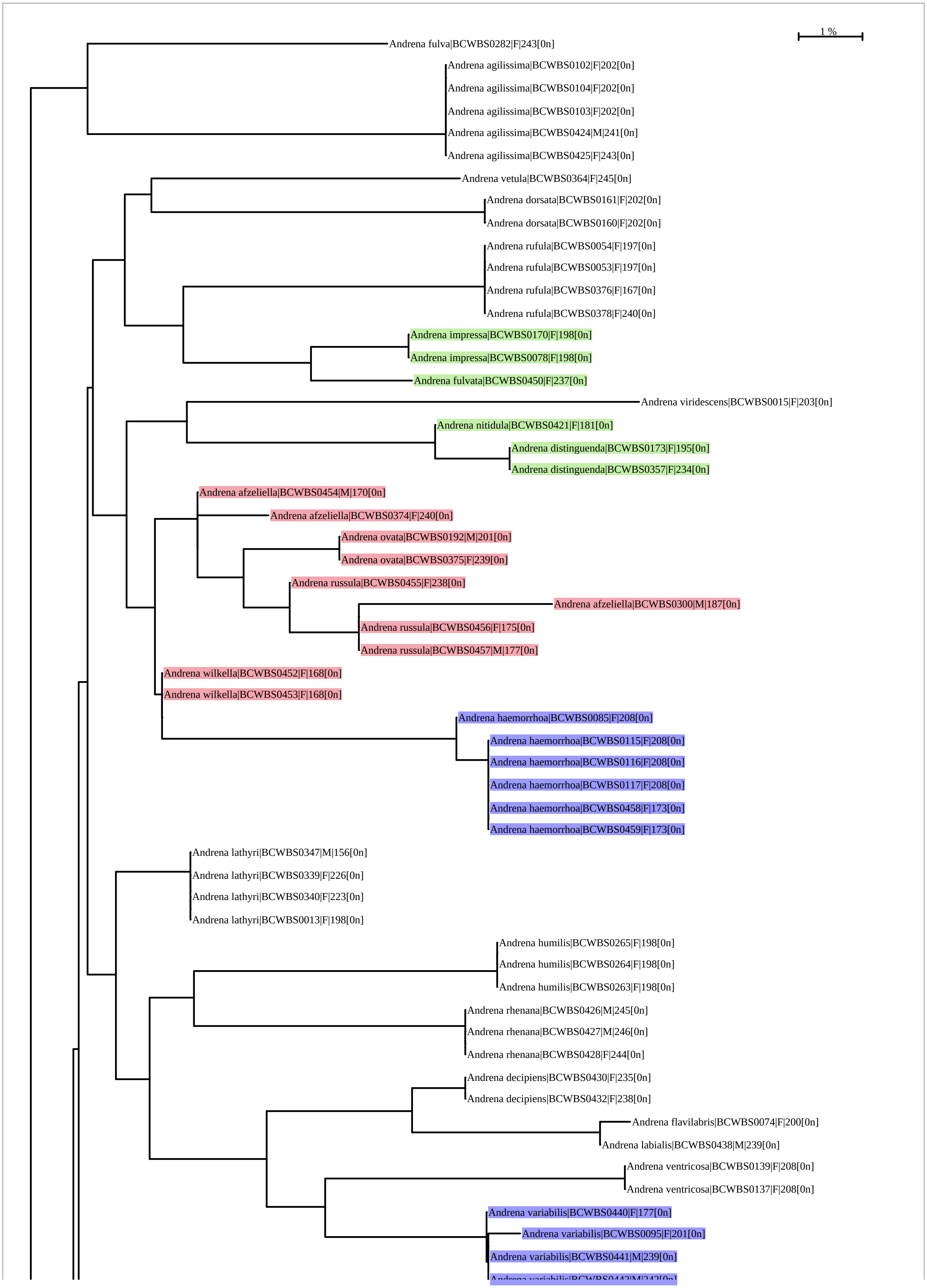
1 %
Andrena fulva|BCWBS0282|F|243[0n]
Andrena agilissima|BCWBS0102|F|202[0n]
Andrena agilissima|BCWBS0104|F|202[0n]
Andrena agilissima|BCWBS0103|F|202[0n]
Andrena agilissima|BCWBS0424|M|241[0n]
Andrena agilissima|BCWBS0425|F|243[0n]
Andrena vetula|BCWBS0364|F|245[0n]
Andrena dorsata|BCWBS0161|F|202[0n]
Andrena dorsata|BCWBS0160|F|202[0n]
Andrena rufula|BCWBS0054|F|197[0n]
Andrena rufula|BCWBS0053|F|197[0n]
Andrena rufula|BCWBS0376|F|167[0n]
Andrena rufula|BCWBS0378|F|240[0n]
Andrena impressa|BCWBS0170|F|198[0n]
Andrena impressa|BCWBS0078|F|198[0n]
Andrena fulvata|BCWBS0450|F|237[0n]
Andrena viridescens|BCWBS0015|F|203[0n]
Andrena nitidula|BCWBS0421|F|181[0n]
Andrena distinguenda|BCWBS0173|F|195[0n]
Andrena distinguenda|BCWBS0357|F|234[0n]
Andrena afzeliella|BCWBS0454|M|170[0n]
Andrena afzeliella|BCWBS0374|F|240[0n]
Andrena ovata|BCWBS0192|M|201[0n]
Andrena ovata|BCWBS0375|F|239[0n]
Andrena russula|BCWBS0455|F|238[0n]
Andrena afzeliella|BCWBS0300|M|187[0n]
Andrena russula|BCWBS0456|F|175[0n]
Andrena russula|BCWBS0457|M|177[0n]
Andrena wilkella|BCWBS0452|F|168[0n]
Andrena wilkella|BCWBS0453|F|168[0n]
Andrena haemorrhoa|BCWBS0085|F|208[0n]
Andrena haemorrhoa|BCWBS0115|F|208[0n]
Andrena haemorrhoa|BCWBS0116|F|208[0n]
Andrena haemorrhoa|BCWBS0117|F|208[0n]
Andrena haemorrhoa|BCWBS0458|F|173[0n]
Andrena haemorrhoa|BCWBS0459|F|173[0n]
Andrena lathyri|BCWBS0347|M|156[0n]
Andrena lathyri|BCWBS0339|F|226[0n]
Andrena lathyri|BCWBS0340|F|223[0n]
Andrena lathyri|BCWBS0013|F|198[0n]
Andrena humilis|BCWBS0265|F|198[0n]
Andrena humilis|BCWBS0264|F|198[0n]
Andrena humilis|BCWBS0263|F|198[0n]
Andrena rhenana|BCWBS0426|M|245[0n]
Andrena rhenana|BCWBS0427|M|246[0n]
Andrena rhenana|BCWBS0428|F|244[0n]
Andrena decipiens|BCWBS0430|F|235[0n]
Andrena decipiens|BCWBS0432|F|238[0n]
Andrena flavilabris|BCWBS0074|F|200[0n]
Andrena labialis|BCWBS0438|M|239[0n]
Andrena ventricosa|BCWBS0139|F|208[0n]
Andrena ventricosa|BCWBS0137|F|208[0n]
Andrena variabilis|BCWBS0440|F|177[0n]
Andrena variabilis|BCWBS0095|F|201[0n]
Andrena variabilis|BCWBS0441|M|239[0n]

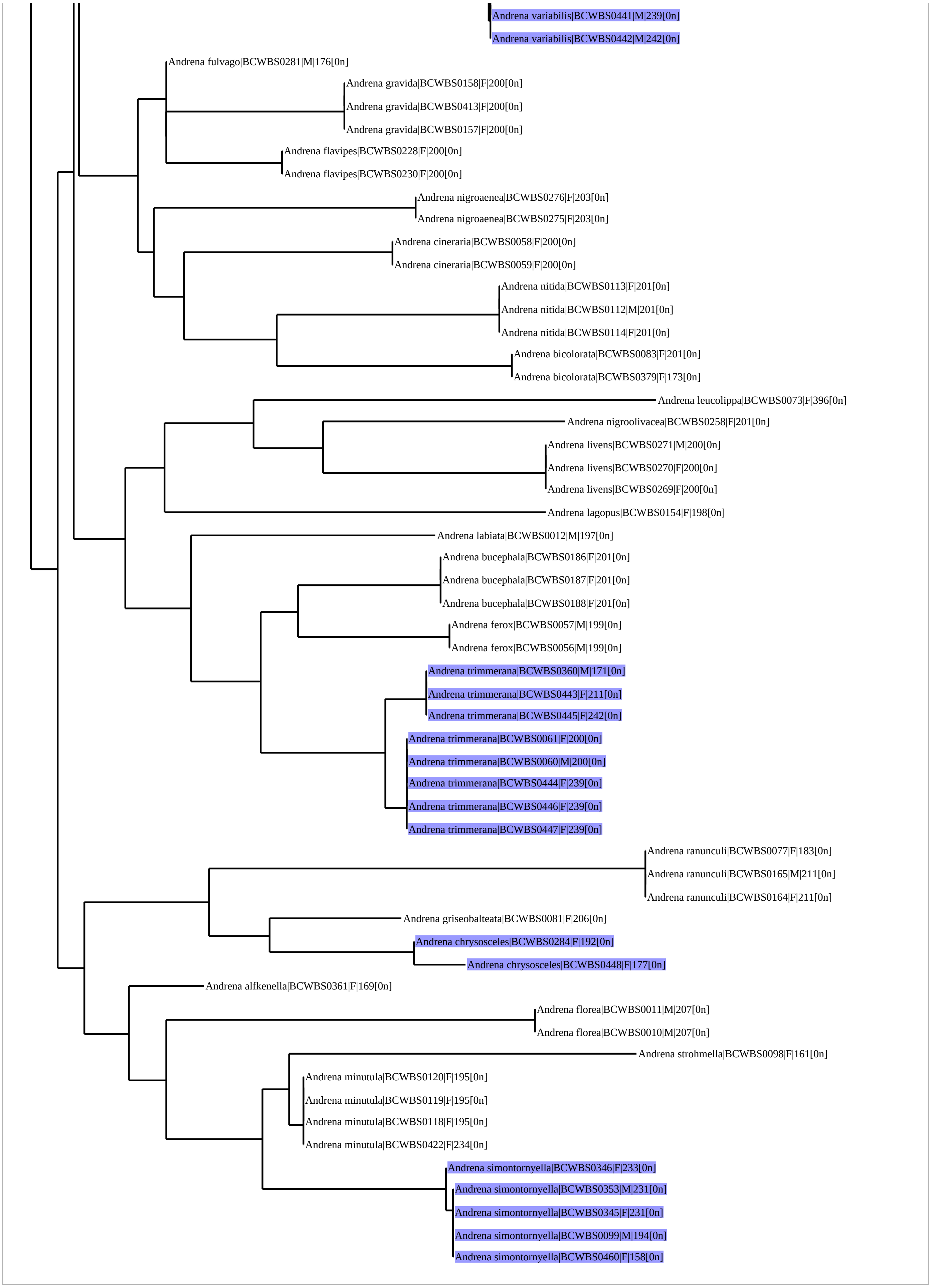
Andrena variabilis|BCWBS0441|M|239[0n]
Andrena variabilis|BCWBS0442|M|242[0n]
Andrena fulvago|BCWBS0281|M|176[0n]
Andrena gravida|BCWBS0158|F|200[0n]
Andrena gravida|BCWBS0413|F|200[0n]
Andrena gravida|BCWBS0157|F|200[0n]
Andrena flavipes|BCWBS0228|F|200[0n]
Andrena flavipes|BCWBS0230|F|200[0n]
Andrena nigroaenea|BCWBS0276|F|203[0n]
Andrena nigroaenea|BCWBS0275|F|203[0n]
Andrena cineraria|BCWBS0058|F|200[0n]
Andrena cineraria|BCWBS0059|F|200[0n]
Andrena nitida|BCWBS0113|F|201[0n]
Andrena nitida|BCWBS0112|M|201[0n]
Andrena nitida|BCWBS0114|F|201[0n]
Andrena bicolorata|BCWBS0083|F|201[0n]
Andrena bicolorata|BCWBS0379|F|173[0n]
Andrena leucolippa|BCWBS0073|F|396[0n]
Andrena nigroolivacea|BCWBS0258|F|201[0n]
Andrena livens|BCWBS0271|M|200[0n]
Andrena livens|BCWBS0270|F|200[0n]
Andrena livens|BCWBS0269|F|200[0n]
Andrena lagopus|BCWBS0154|F|198[0n]
Andrena labiata|BCWBS0012|M|197[0n]
Andrena bucephala|BCWBS0186|F|201[0n]
Andrena bucephala|BCWBS0187|F|201[0n]
Andrena bucephala|BCWBS0188|F|201[0n]
Andrena ferox|BCWBS0057|M|199[0n]
Andrena ferox|BCWBS0056|M|199[0n]
Andrena trimmerana|BCWBS0360|M|171[0n]
Andrena trimmerana|BCWBS0443|F|211[0n]
Andrena trimmerana|BCWBS0445|F|242[0n]
Andrena trimmerana|BCWBS0061|F|200[0n]
Andrena trimmerana|BCWBS0060|M|200[0n]
Andrena trimmerana|BCWBS0444|F|239[0n]
Andrena trimmerana|BCWBS0446|F|239[0n]
Andrena trimmerana|BCWBS0447|F|239[0n]
Andrena ranunculi|BCWBS0077|F|183[0n]
Andrena ranunculi|BCWBS0165|M|211[0n]
Andrena ranunculi|BCWBS0164|F|211[0n]
Andrena griseobalteata|BCWBS0081|F|206[0n]
Andrena chrysosceles|BCWBS0284|F|192[0n]
Andrena chrysosceles|BCWBS0448|F|177[0n]
Andrena alfkenella|BCWBS0361|F|169[0n]
Andrena florea|BCWBS0011|M|207[0n]
Andrena florea|BCWBS0010|M|207[0n]
Andrena strohmella|BCWBS0098|F|161[0n]
Andrena minutula|BCWBS0120|F|195[0n]
Andrena minutula|BCWBS0119|F|195[0n]
Andrena minutula|BCWBS0118|F|195[0n]
Andrena minutula|BCWBS0422|F|234[0n]
Andrena simontornyella|BCWBS0346|F|233[0n]
Andrena simontornyella|BCWBS0353|M|231[0n]
Andrena simontornyella|BCWBS0345|F|231[0n]
Andrena simontornyella|BCWBS0099|M|194[0n]
Andrena simontornyella|BCWBS0460|F|158[0n]
