# Supplementary material for: Building a reliable 16S mini-barcode library of wild bees from Occitania, south-west of France
Source: Biodivers Data J. 2025 Jan 7;13:e137540. doi: 10.3897/BDJ.12.e137540 (PMC11733625; doi:10.3897/BDJ.12.e137540)
Supplement: Supplementary material 9 — Apidae Tree [file bdj-13-e137540-s009.pdf]

BOLD TaxonID Tree

Title	:	Tree Result - DS-APID16
Date	:	03-Sep-2024
Data Type	:	Nucleotide
Distance Model	:	Kimura 2 Parameter
Marker	:	16S
Colourization	:	[blue]=Stop Codons [red]=Contamination or misidentification
Label	:	Sample ID
Label	:	Taxon
Label	:	Sex/Gender
Label	:	Sequence Length
Sequence Count	:	128
Species count	:	49
Genus count	:	6
Family count	:	1
Unidentified	:	0
BIN Count	:	0

Legend :

- Allelic variant
- Non discriminant 16S
- Discriminant 16S for group species

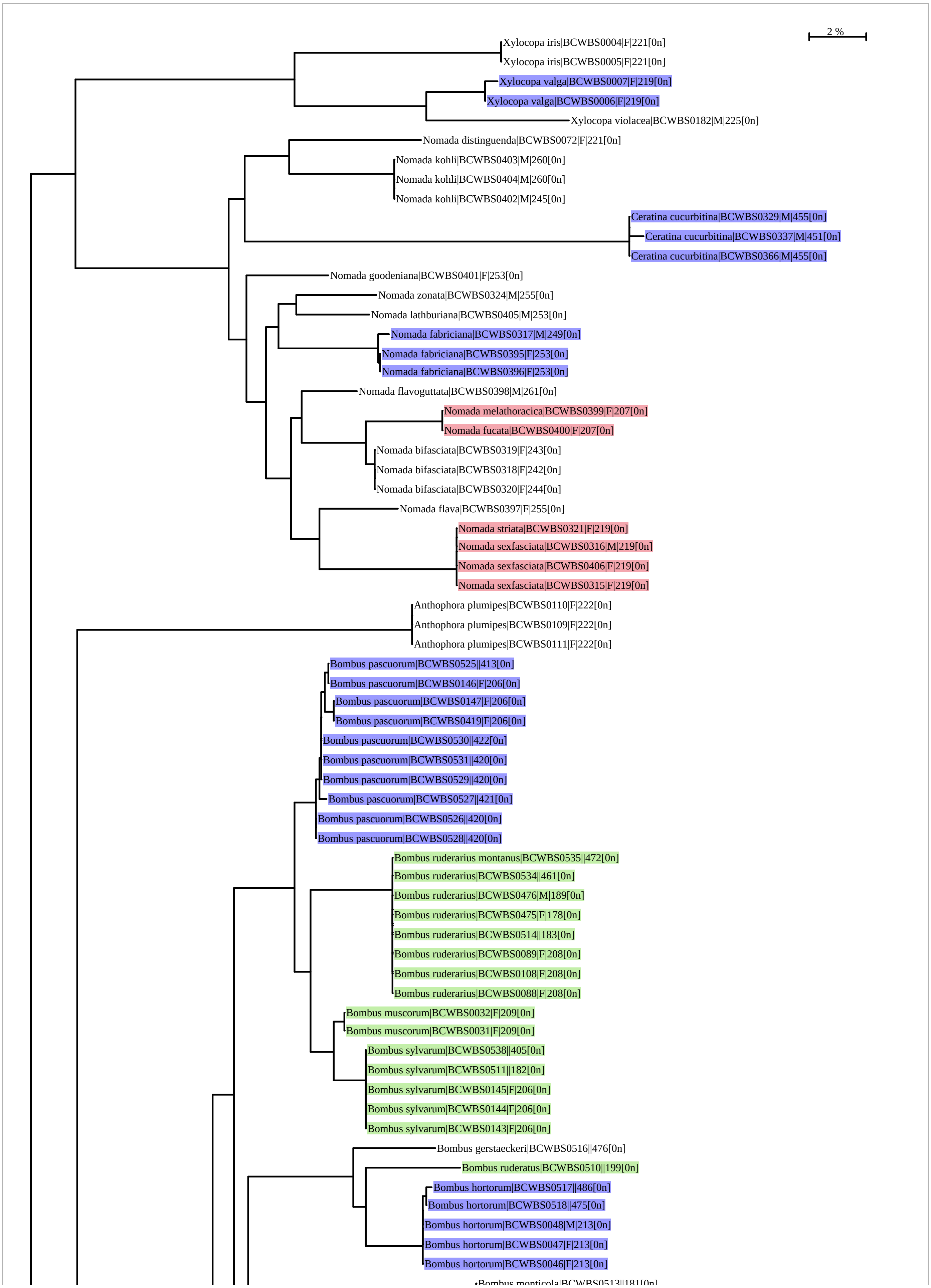
2 %
Xylocopa iris|BCWBS0004|F|221[0n]
Xylocopa iris|BCWBS0005|F|221[0n]
Xylocopa valga|BCWBS0007|F|219[0n]
Xylocopa valga|BCWBS0006|F|219[0n]
Xylocopa violacea|BCWBS0182|M|225[0n]
Nomada distinguenda|BCWBS0072|F|221[0n]
Nomada kohli|BCWBS0403|M|260[0n]
Nomada kohli|BCWBS0404|M|260[0n]
Nomada kohli|BCWBS0402|M|245[0n]
Ceratina cucurbitina|BCWBS0329|M|455[0n]
Ceratina cucurbitina|BCWBS0337|M|451[0n]
Ceratina cucurbitina|BCWBS0366|M|455[0n]
Nomada goodeniana|BCWBS0401|F|253[0n]
Nomada zonata|BCWBS0324|M|255[0n]
Nomada lathburiana|BCWBS0405|M|253[0n]
Nomada fabriciana|BCWBS0317|M|249[0n]
Nomada fabriciana|BCWBS0395|F|253[0n]
Nomada fabriciana|BCWBS0396|F|253[0n]
Nomada flavoguttata|BCWBS0398|M|261[0n]
Nomada melathoracica|BCWBS0399|F|207[0n]
Nomada fucata|BCWBS0400|F|207[0n]
Nomada bifasciata|BCWBS0319|F|243[0n]
Nomada bifasciata|BCWBS0318|F|242[0n]
Nomada bifasciata|BCWBS0320|F|244[0n]
Nomada flava|BCWBS0397|F|255[0n]
Nomada striata|BCWBS0321|F|219[0n]
Nomada sexfasciata|BCWBS0316|M|219[0n]
Nomada sexfasciata|BCWBS0406|F|219[0n]
Nomada sexfasciata|BCWBS0315|F|219[0n]
Anthophora plumipes|BCWBS0110|F|222[0n]
Anthophora plumipes|BCWBS0109|F|222[0n]
Anthophora plumipes|BCWBS0111|F|222[0n]
Bombus pascuorum|BCWBS0525||413[0n]
Bombus pascuorum|BCWBS0146|F|206[0n]
Bombus pascuorum|BCWBS0147|F|206[0n]
Bombus pascuorum|BCWBS0419|F|206[0n]
Bombus pascuorum|BCWBS0530||422[0n]
Bombus pascuorum|BCWBS0531||420[0n]
Bombus pascuorum|BCWBS0529||420[0n]
Bombus pascuorum|BCWBS0527||421[0n]
Bombus pascuorum|BCWBS0526||420[0n]
Bombus pascuorum|BCWBS0528||420[0n]
Bombus ruderarius montanus|BCWBS0535||472[0n]
Bombus ruderarius|BCWBS0534||461[0n]
Bombus ruderarius|BCWBS0476|M|189[0n]
Bombus ruderarius|BCWBS0475|F|178[0n]
Bombus ruderarius|BCWBS0514||183[0n]
Bombus ruderarius|BCWBS0089|F|208[0n]
Bombus ruderarius|BCWBS0108|F|208[0n]
Bombus ruderarius|BCWBS0088|F|208[0n]
Bombus muscorum|BCWBS0032|F|209[0n]
Bombus muscorum|BCWBS0031|F|209[0n]
Bombus sylvarum|BCWBS0538||405[0n]
Bombus sylvarum|BCWBS0511||182[0n]
Bombus sylvarum|BCWBS0145|F|206[0n]
Bombus sylvarum|BCWBS0144|F|206[0n]
Bombus sylvarum|BCWBS0143|F|206[0n]
Bombus gerstaeckeri|BCWBS0516||476[0n]
Bombus ruderatus|BCWBS0510||199[0n]
Bombus hortorum|BCWBS0517||486[0n]
Bombus hortorum|BCWBS0518||475[0n]
Bombus hortorum|BCWBS0048|M|213[0n]
Bombus hortorum|BCWBS0047|F|213[0n]
Bombus hortorum|BCWBS0046|F|213[0n]
Bombus monticola|BCWBS0513||181[0n]

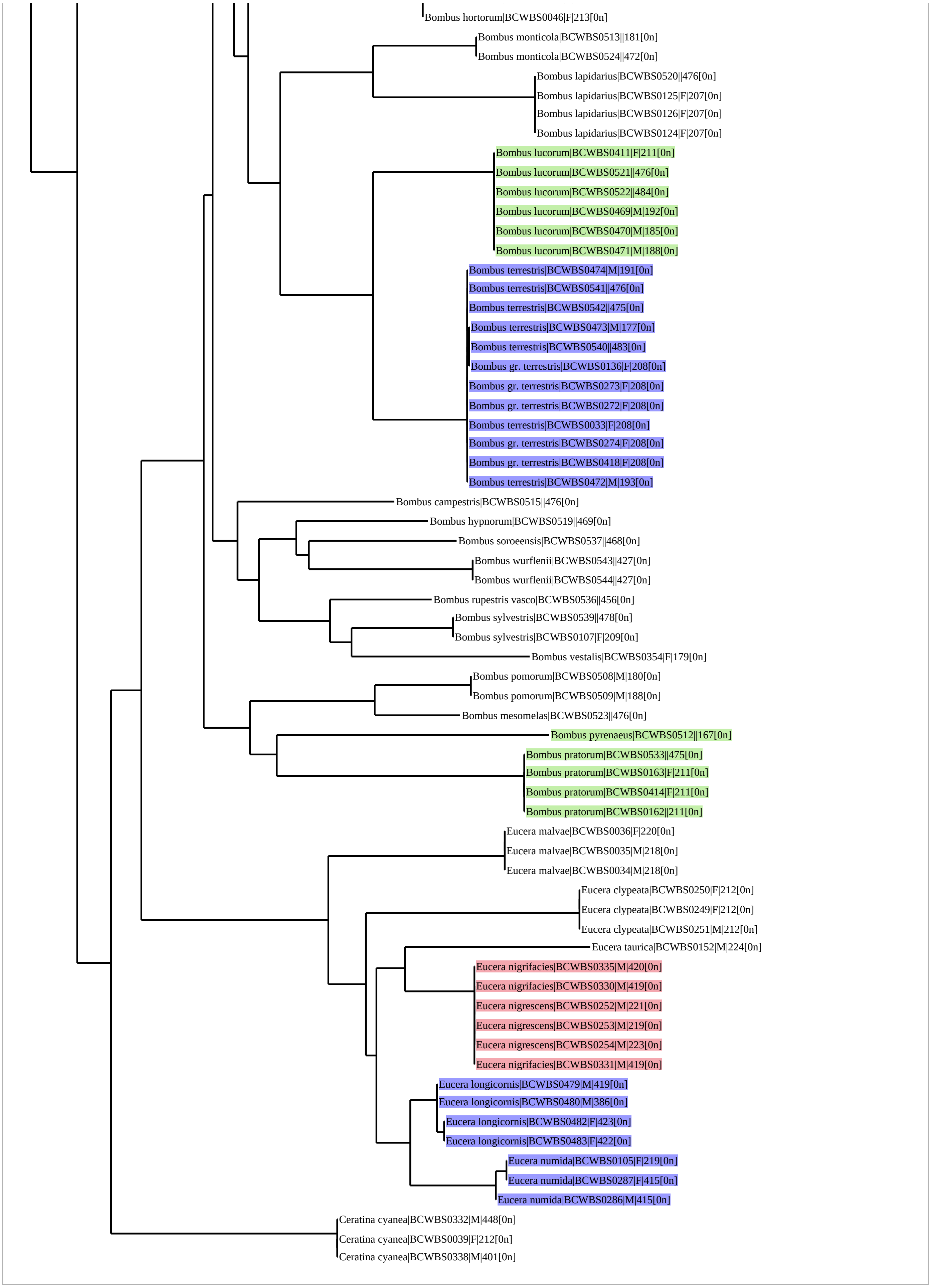
Bombus hortorum|BCWBS0046|F|213[0n]
Bombus monticola|BCWBS0513||181[0n]
Bombus monticola|BCWBS0524||472[0n]
Bombus lapidarius|BCWBS0520||476[0n]
Bombus lapidarius|BCWBS0125|F|207[0n]
Bombus lapidarius|BCWBS0126|F|207[0n]
Bombus lapidarius|BCWBS0124|F|207[0n]
Bombus lucorum|BCWBS0411|F|211[0n]
Bombus lucorum|BCWBS0521||476[0n]
Bombus lucorum|BCWBS0522||484[0n]
Bombus lucorum|BCWBS0469|M|192[0n]
Bombus lucorum|BCWBS0470|M|185[0n]
Bombus lucorum|BCWBS0471|M|188[0n]
Bombus terrestris|BCWBS0474|M|191[0n]
Bombus terrestris|BCWBS0541||476[0n]
Bombus terrestris|BCWBS0542||475[0n]
Bombus terrestris|BCWBS0473|M|177[0n]
Bombus terrestris|BCWBS0540||483[0n]
Bombus gr. terrestris|BCWBS0136|F|208[0n]
Bombus gr. terrestris|BCWBS0273|F|208[0n]
Bombus gr. terrestris|BCWBS0272|F|208[0n]
Bombus terrestris|BCWBS0033|F|208[0n]
Bombus gr. terrestris|BCWBS0274|F|208[0n]
Bombus gr. terrestris|BCWBS0418|F|208[0n]
Bombus terrestris|BCWBS0472|M|193[0n]
Bombus campestris|BCWBS0515||476[0n]
Bombus hypnorum|BCWBS0519||469[0n]
Bombus soroeensis|BCWBS0537||468[0n]
Bombus wurflenii|BCWBS0543||427[0n]
Bombus wurflenii|BCWBS0544||427[0n]
Bombus rupestris vasco|BCWBS0536||456[0n]
Bombus sylvestris|BCWBS0539||478[0n]
Bombus sylvestris|BCWBS0107|F|209[0n]
Bombus vestalis|BCWBS0354|F|179[0n]
Bombus pomorum|BCWBS0508|M|180[0n]
Bombus pomorum|BCWBS0509|M|188[0n]
Bombus mesomelas|BCWBS0523||476[0n]
Bombus pyrenaeus|BCWBS0512||167[0n]
Bombus pratorum|BCWBS0533||475[0n]
Bombus pratorum|BCWBS0163|F|211[0n]
Bombus pratorum|BCWBS0414|F|211[0n]
Bombus pratorum|BCWBS0162||211[0n]
Eucera malvae|BCWBS0036|F|220[0n]
Eucera malvae|BCWBS0035|M|218[0n]
Eucera malvae|BCWBS0034|M|218[0n]
Eucera clypeata|BCWBS0250|F|212[0n]
Eucera clypeata|BCWBS0249|F|212[0n]
Eucera clypeata|BCWBS0251|M|212[0n]
Eucera taurica|BCWBS0152|M|224[0n]
Eucera nigrifacies|BCWBS0335|M|420[0n]
Eucera nigrifacies|BCWBS0330|M|419[0n]
Eucera nigrescens|BCWBS0252|M|221[0n]
Eucera nigrescens|BCWBS0253|M|219[0n]
Eucera nigrescens|BCWBS0254|M|223[0n]
Eucera nigrifacies|BCWBS0331|M|419[0n]
Eucera longicornis|BCWBS0479|M|419[0n]
Eucera longicornis|BCWBS0480|M|386[0n]
Eucera longicornis|BCWBS0482|F|423[0n]
Eucera longicornis|BCWBS0483|F|422[0n]
Eucera numida|BCWBS0105|F|219[0n]
Eucera numida|BCWBS0287|F|415[0n]
Eucera numida|BCWBS0286|M|415[0n]
Ceratina cyanea|BCWBS0332|M|448[0n]
Ceratina cyanea|BCWBS0039|F|212[0n]
Ceratina cyanea|BCWBS0338|M|401[0n]
