# Supplementary material for: Building a reliable 16S mini-barcode library of wild bees from Occitania, south-west of France
Source: Biodivers Data J. 2025 Jan 7;13:e137540. doi: 10.3897/BDJ.12.e137540 (PMC11733625; doi:10.3897/BDJ.12.e137540)
Supplement: Supplementary material 10 — Colletidae Tree [file bdj-13-e137540-s010.pdf]

BOLD TaxonID Tree

Title	:	Tree Result - DS-COLLET16
Date	:	03-Sep-2024
Data Type	:	Nucleotide
Distance Model	:	Kimura 2 Parameter
Marker	:	16S
Colourization	:	[blue]=Stop Codons [red]=Contamination or misidentification
Label	:	Sample ID
Label	:	Taxon
Label	:	Sex/Gender
Label	:	Sequence Length
Sequence Count	:	9
Species count	:	6
Genus count	:	2
Family count	:	1
Unidentified	:	0
BIN Count	:	0

Legend :

- Allelic variant
- Non discriminant 16S
- Discriminant 16S for group species

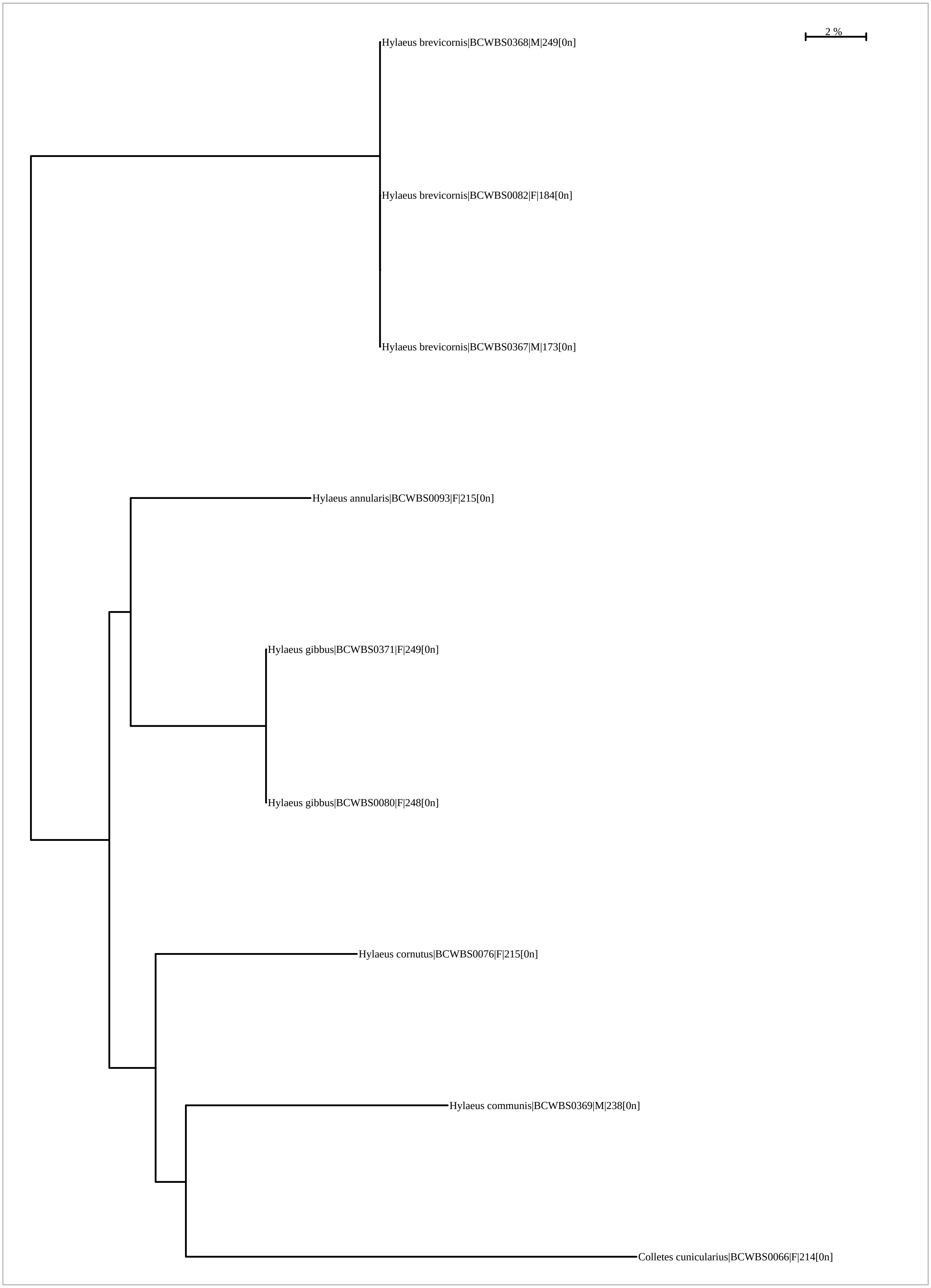
2 %
Hylaeus brevicornis|BCWBS0368|M|249[0n]
Hylaeus brevicornis|BCWBS0082|F|184[0n]
Hylaeus brevicornis|BCWBS0367|M|173[0n]
Hylaeus annularis|BCWBS0093|F|215[0n]
Hylaeus gibbus|BCWBS0371|F|249[0n]
Hylaeus gibbus|BCWBS0080|F|248[0n]
Hylaeus cornutus|BCWBS0076|F|215[0n]
Hylaeus communis|BCWBS0369|M|238[0n]
Colletes cunicularius|BCWBS0066|F|214[0n]
