# Supplementary material for: Building a reliable 16S mini-barcode library of wild bees from Occitania, south-west of France
Source: Biodivers Data J. 2025 Jan 7;13:e137540. doi: 10.3897/BDJ.12.e137540 (PMC11733625; doi:10.3897/BDJ.12.e137540)
Supplement: Supplementary material 11 — Megachilidae Tree [file bdj-13-e137540-s011.pdf]

BOLD TaxonID Tree

Title : Tree Result - DS-MEGA16
Date : 03-Sep-2024
Data Type : Nucleotide
Distance Model : Kimura 2 Parameter
Marker : 16S
Colourization : [blue]=Stop Codons [red]=Contamination or misidentification

Label : Sample ID
Label : Taxon
Label : Sex/Gender
Label : Sequence Length

Sequence Count : 19
Species count : 9
Genus count : 3
Family count : 1
Unidentified : 0

BIN Count : 0

Legend :

- Allelic variant
- Non discriminant 16S
- Discriminant 16S for group species

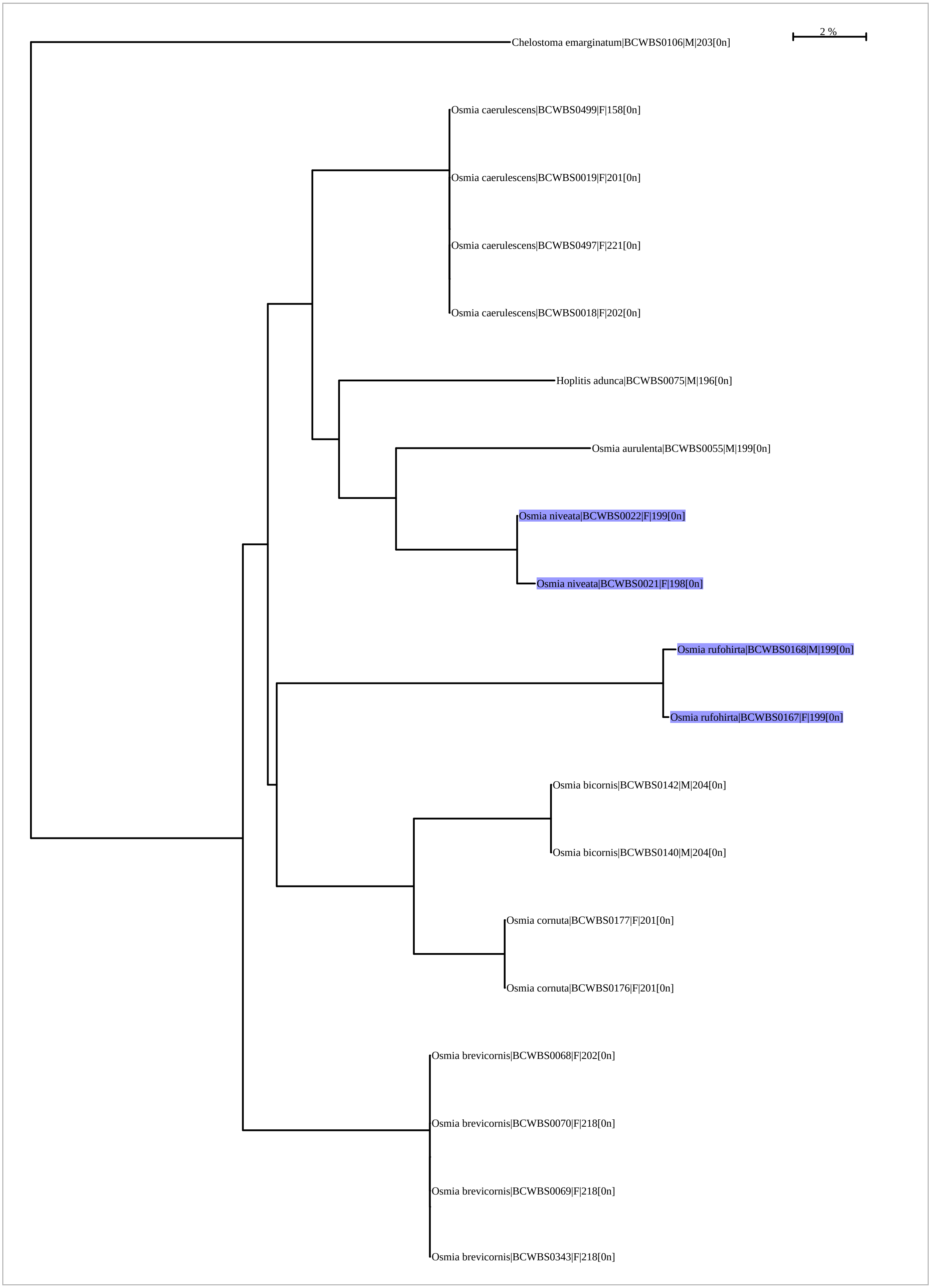
2 %
Chelostoma emarginatum|BCWBS0106|M|203[0n]
Osmia caerulescens|BCWBS0499|F|158[0n]
Osmia caerulescens|BCWBS0019|F|201[0n]
Osmia caerulescens|BCWBS0497|F|221[0n]
Osmia caerulescens|BCWBS0018|F|202[0n]
Hoplitis adunca|BCWBS0075|M|196[0n]
Osmia aurulenta|BCWBS0055|M|199[0n]
Osmia niveata|BCWBS0022|F|199[0n]
Osmia niveata|BCWBS0021|F|198[0n]
Osmia rufohirta|BCWBS0168|M|199[0n]
Osmia rufohirta|BCWBS0167|F|199[0n]
Osmia bicornis|BCWBS0142|M|204[0n]
Osmia bicornis|BCWBS0140|M|204[0n]
Osmia cornuta|BCWBS0177|F|201[0n]
Osmia cornuta|BCWBS0176|F|201[0n]
Osmia brevicornis|BCWBS0068|F|202[0n]
Osmia brevicornis|BCWBS0070|F|218[0n]
Osmia brevicornis|BCWBS0069|F|218[0n]
Osmia brevicornis|BCWBS0343|F|218[0n]
