# Supplementary material for: Building a reliable 16S mini-barcode library of wild bees from Occitania, south-west of France
Source: Biodivers Data J. 2025 Jan 7;13:e137540. doi: 10.3897/BDJ.12.e137540 (PMC11733625; doi:10.3897/BDJ.12.e137540)
Supplement: Supplementary material 12 — Halictidae Tree [file bdj-13-e137540-s012.pdf]

BOLD TaxonID Tree

```
Title           : Tree Result - DS-HALIC16
Date            : 03-Sep-2024
Data Type       : Nucleotide
Distance Model  : Kimura 2 Parameter
Marker          : 16S
Colourization   : [blue]=Stop Codons [red]=Contamination or misidentification

Label           : Sample ID
Label           : Taxon
Label           : Sex/Gender
Label           : Sequence Length

Sequence Count  : 81
Species count   : 37
Genus count     : 4
Family count    : 1
Unidentified    : 0

BIN Count       : 0
```

Legend :

- Allelic variant
- Non discriminant 16S
- Discriminant 16S for group species

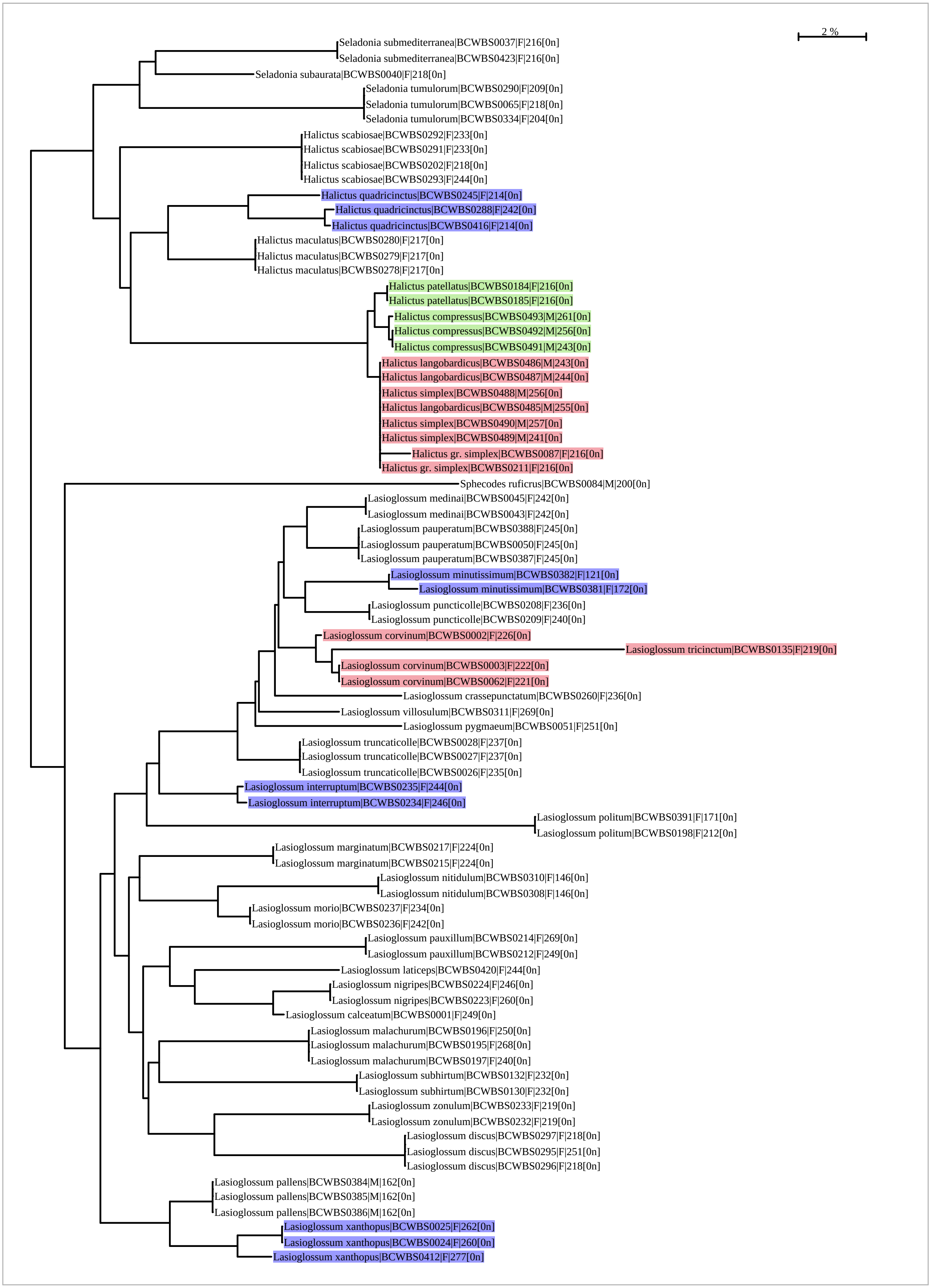
2 %
Seladonia submediterranea|BCWBS0037|F|216[0n]
Seladonia submediterranea|BCWBS0423|F|216[0n]
Seladonia subaurata|BCWBS0040|F|218[0n]
Seladonia tumulorum|BCWBS0290|F|209[0n]
Seladonia tumulorum|BCWBS0065|F|218[0n]
Seladonia tumulorum|BCWBS0334|F|204[0n]
Halictus scabiosae|BCWBS0292|F|233[0n]
Halictus scabiosae|BCWBS0291|F|233[0n]
Halictus scabiosae|BCWBS0202|F|218[0n]
Halictus scabiosae|BCWBS0293|F|244[0n]
Halictus quadricinctus|BCWBS0245|F|214[0n]
Halictus quadricinctus|BCWBS0288|F|242[0n]
Halictus quadricinctus|BCWBS0416|F|214[0n]
Halictus maculatus|BCWBS0280|F|217[0n]
Halictus maculatus|BCWBS0279|F|217[0n]
Halictus maculatus|BCWBS0278|F|217[0n]
Halictus patellatus|BCWBS0184|F|216[0n]
Halictus patellatus|BCWBS0185|F|216[0n]
Halictus compressus|BCWBS0493|M|261[0n]
Halictus compressus|BCWBS0492|M|256[0n]
Halictus compressus|BCWBS0491|M|243[0n]
Halictus langobardicus|BCWBS0486|M|243[0n]
Halictus langobardicus|BCWBS0487|M|244[0n]
Halictus simplex|BCWBS0488|M|256[0n]
Halictus langobardicus|BCWBS0485|M|255[0n]
Halictus simplex|BCWBS0490|M|257[0n]
Halictus simplex|BCWBS0489|M|241[0n]
Halictus gr. simplex|BCWBS0087|F|216[0n]
Halictus gr. simplex|BCWBS0211|F|216[0n]
Sphecodes ruficrus|BCWBS0084|M|200[0n]
Lasioglossum medinai|BCWBS0045|F|242[0n]
Lasioglossum medinai|BCWBS0043|F|242[0n]
Lasioglossum pauperatum|BCWBS0388|F|245[0n]
Lasioglossum pauperatum|BCWBS0050|F|245[0n]
Lasioglossum pauperatum|BCWBS0387|F|245[0n]
Lasioglossum minutissimum|BCWBS0382|F|121[0n]
Lasioglossum minutissimum|BCWBS0381|F|172[0n]
Lasioglossum puncticolle|BCWBS0208|F|236[0n]
Lasioglossum puncticolle|BCWBS0209|F|240[0n]
Lasioglossum corvinum|BCWBS0002|F|226[0n]
Lasioglossum tricinctum|BCWBS0135|F|219[0n]
Lasioglossum corvinum|BCWBS0003|F|222[0n]
Lasioglossum corvinum|BCWBS0062|F|221[0n]
Lasioglossum crassepunctatum|BCWBS0260|F|236[0n]
Lasioglossum villosulum|BCWBS0311|F|269[0n]
Lasioglossum pygmaeum|BCWBS0051|F|251[0n]
Lasioglossum truncaticolle|BCWBS0028|F|237[0n]
Lasioglossum truncaticolle|BCWBS0027|F|237[0n]
Lasioglossum truncaticolle|BCWBS0026|F|235[0n]
Lasioglossum interruptum|BCWBS0235|F|244[0n]
Lasioglossum interruptum|BCWBS0234|F|246[0n]
Lasioglossum politum|BCWBS0391|F|171[0n]
Lasioglossum politum|BCWBS0198|F|212[0n]
Lasioglossum marginatum|BCWBS0217|F|224[0n]
Lasioglossum marginatum|BCWBS0215|F|224[0n]
Lasioglossum nitidulum|BCWBS0310|F|146[0n]
Lasioglossum nitidulum|BCWBS0308|F|146[0n]
Lasioglossum morio|BCWBS0237|F|234[0n]
Lasioglossum morio|BCWBS0236|F|242[0n]
Lasioglossum pauxillum|BCWBS0214|F|269[0n]
Lasioglossum pauxillum|BCWBS0212|F|249[0n]
Lasioglossum laticeps|BCWBS0420|F|244[0n]
Lasioglossum nigripes|BCWBS0224|F|246[0n]
Lasioglossum nigripes|BCWBS0223|F|260[0n]
Lasioglossum calceatum|BCWBS0001|F|249[0n]
Lasioglossum malachurum|BCWBS0196|F|250[0n]
Lasioglossum malachurum|BCWBS0195|F|268[0n]
Lasioglossum malachurum|BCWBS0197|F|240[0n]
Lasioglossum subhirtum|BCWBS0132|F|232[0n]
Lasioglossum subhirtum|BCWBS0130|F|232[0n]
Lasioglossum zonulum|BCWBS0233|F|219[0n]
Lasioglossum zonulum|BCWBS0232|F|219[0n]
Lasioglossum discus|BCWBS0297|F|218[0n]
Lasioglossum discus|BCWBS0295|F|251[0n]
Lasioglossum discus|BCWBS0296|F|218[0n]
Lasioglossum pallens|BCWBS0384|M|162[0n]
Lasioglossum pallens|BCWBS0385|M|162[0n]
Lasioglossum pallens|BCWBS0386|M|162[0n]
Lasioglossum xanthopus|BCWBS0025|F|262[0n]
Lasioglossum xanthopus|BCWBS0024|F|260[0n]
Lasioglossum xanthopus|BCWBS0412|F|277[0n]
